# Osteoblast Attachment on Bioactive Glass Air Particle Abrasion-Induced Calcium Phosphate Coating

**DOI:** 10.3390/bioengineering11010074

**Published:** 2024-01-12

**Authors:** Faleh Abushahba, Elina Kylmäoja, Nagat Areid, Leena Hupa, Pekka K. Vallittu, Juha Tuukkanen, Timo Närhi

**Affiliations:** 1 Department of Biomaterials Science and Turku Clinical Biomaterial Center—TCBC, Institute of Dentistry, University of Turku, 20520 Turku, Finland; pekval@utu.fi; 2 Department of Prosthetic Dentistry and Stomatognathic Physiology, Institute of Dentistry, University of Turku, 20520 Turku, Finland; nmaare@utu.fi; 3 Department of Restorative Dentistry and Periodontology, Faculty of Dentistry, Libyan International Medical University (LIMU), Benghazi 339P+62Q, Libya; 4 Department of Anatomy and Cell Biology, Research Unit of Translational Medicine, Medical Research Center, University of Oulu, 90014 Oulu, Finland; elina.kylmaoja@oulu.fi (E.K.); juha.tuukkanen@oulu.fi (J.T.); 5 Johan Gadolin Process Chemistry Center, Åbo Akademi University, Henriksgatan 2, 20500 Turku, Finland; leena.hupa@abo.fi; 6 The Wellbeing Service County Southwest Finland, 20521 Turku, Finland

**Keywords:** biofilm, biomineralization, implant, osteoblast, peri-implantitis, *S. mutans*, titanium, zinc

## Abstract

Air particle abrasion (APA) using bioactive glass (BG) effectively decontaminates titanium (Ti) surface biofilms and the retained glass particles on the abraded surfaces impart potent antibacterial properties against various clinically significant pathogens. The objective of this study was to investigate the effect of BG APA and simulated body fluid (SBF) immersion of sandblasted and acid-etched (SA) Ti surfaces on osteoblast cell viability. Another goal was to study the antibacterial effect against *Streptococcus mutans*. Square-shaped 10 mm diameter Ti substrates (*n* = 136) were SA by grit blasting with aluminum oxide particles, then acid-etching in an HCl-H_2_SO_4_ mixture. The SA substrates (*n* = 68) were used as non-coated controls (NC-SA). The test group (*n* = 68) was further subjected to APA using experimental zinc-containing BG (Zn4) and then mineralized in SBF for 14 d (Zn4-CaP). Surface roughness, contact angle, and surface free energy (SFE) were calculated on test and control surfaces. In addition, the topography and chemistry of substrate surfaces were also characterized. Osteoblastic cell viability and focal adhesion were also evaluated and compared to glass slides as an additional control. The antibacterial effect of Zn4-CaP was also assessed against *S. mutans*. After immersion in SBF, a mineralized zinc-containing Ca-P coating was formed on the SA substrates. The Zn4-CaP coating resulted in a significantly lower Ra surface roughness value (2.565 μm; *p* < 0.001), higher wettability (13.35°; *p* < 0.001), and higher total SFE (71.13; *p* < 0.001) compared to 3.695 μm, 77.19° and 40.43 for the NC-SA, respectively. APA using Zn4 can produce a zinc-containing calcium phosphate coating that demonstrates osteoblast cell viability and focal adhesion comparable to that on NC-SA or glass slides. Nevertheless, the coating had no antibacterial effect against *S. mutans.*

## 1. Introduction

Peri-implantitis (PI) is an inflammatory condition caused by a complex biofilm structure on implant surfaces that leads to an inflammatory response, resulting in progressive and irreversible implant-supporting tissue loss [1]. Therefore, the ideal PI therapy should focus on implant surface decontamination followed by bone regeneration of lost tissues. Nevertheless, the design of implants and the roughness of their surfaces enable plaque accumulation and hinder the efficiency of mechanical debridement, resulting in unsatisfactory treatment outcomes [2,3].

Various conventional treatment methods, such as curettes, sonic and ultrasonic scalers, carbon tips, laser, implantoplasty, and air particle abrasion (APA), have been used for implant surface decontamination [2,3,4,5]. APA is the most efficient method in biofilm removal, and it does not cause implant surface damage compared to other mechanical debridement methods, as demonstrated by Keim et al. [6] Nevertheless, the primary drawback of APA lies in its inability to fully restore the biocompatibility of the abraded surface [7]. Furthermore, the abrasive material could either positively or negatively affect the healing process in the vicinity of the decontaminated implant. Using bioactive glasses (BG) in APA has been shown to effectively decontaminate bacterial biofilms from titanium (Ti) surfaces and impart antibacterial properties to abraded surfaces [8,9,10]. Furthermore, APA of Ti surfaces using BG promotes the adhesion and proliferation of pre-osteoblastic MC3T3-E1 cells [11].

BG gradually dissolves in simulated body fluid (SBF) due to a series of reactions initiated at the glass surface. First, sodium (Na^+^) and calcium (Ca^2+^) ions in the glass surface are leached by hydration or ion exchange with (H^+^), leading to an elevated concentration of hydroxide (OH^−^) and the local alkaline environment, and consequently, increased pH and osmotic pressure. The rise in the pH and osmotic pressure are suggested as the mechanism underlying the antibacterial properties of BG [12,13,14,15,16,17]. In further reactions, soluble silica is released into the solution, followed by the condensation and repolymerization of an orthosilicic acid gel on the surface, which serves as a matrix for hydroxyapatite (HA) formation [18]. The gradual formation of a dual surface comprising an inner silica-rich and an outer HA layer is likely to change the dissolution kinetics of the glass, thereby influencing the biological responses [19].

Various methods are used to deposit a calcium phosphate (Ca-P) layer on the Ti surface to enhance the adhesion between bone and metal, such as physical and chemical vapor deposition and implantation techniques [20,21]. Despite the good osteoconductive properties, these methods encounter several issues, such as brittleness, flaking, and delamination due to the low cohesive strength and elevated processing temperature, leading to inhomogeneity of the chemical composition [22,23]. Plasma electrolytic oxidation is another method to produce good adhesion between the Ca-P layer and metal surfaces [24]. Also, atomic layer deposition can coat the titanium with a thin HA layer with good adhesion strength to the Ti surface [25].

The Bioglass^®^45S5 has undergone modifications by the addition of small ratios of biologically active elements such as copper (Cu), magnesium (Mg), strontium (Sr), silver (Ag), and zinc (Zn) to improve therapeutic behavior and impart specific biological functionalities. Ag-dopped borosilicate glass has shown a rapid HA layer formation and significant antibacterial efficacy against *Escherichia coli* and *Staphylococcus aureus* [26]. However, despite its favorable antibacterial effect, high Ag concentration in the glass composition can cause cytotoxicity and inhibit cell proliferation [27]. Zn is also an antimicrobial element that demonstrates strong antibacterial effects against many clinically significant pathogens by various mechanisms. The Zn^2+^ ions have the ability to permeate the bacterial cell membrane and generate reactive oxygen species (ROS), causing bacterial death. Furthermore, it disturbs protein synthesis and interferes with the DNA replication process of bacterial cells [28]. Additionally, the Zn^2+^ ion plays a crucial role in the proliferation and differentiation of osteoblast cells, hinders osteoclastic cell activity, and contributes significantly to the cell’s growth factors expression [29,30,31].

A previous study demonstrated that after APA of Ti surface with BG, remnant glass particles firmly attached to the surface could still produce an extended antibacterial effect [8]. Further, Koller and co-workers have shown that the detached Bioglass^®^45S5 particles from APA of the Ti surface produced an HA layer that resembles the mineral phase of human bone [32]. However, it is not known whether the retained glass particles on the Ti surface during the reactions can produce a biologically active HA layer and promote osteoblast attachment. Similarly, the potential antibacterial activity of the in vitro treated surface has not been established.

Therefore, this study aimed to investigate the potential HA layer formation in SBF on a Ti surface abraded by particles of an experimental BG. The final goal was to study the osteoblast adhesion and viability as well as the antibacterial effects against *Streptococcus mutans* after SBF immersion. Our working hypothesis was that the APA procedure results in embedding a certain fraction of the BG particles onto the SA Ti surface. The firmly attached glass particles will form a biologically active HA layer that enables osteoblast cell adhesion and proliferation.

## 2. Materials and Methods

### 2.1. Preparation of Titanium Substrates

The Ti substrate was prepared based on the method described earlier [9]. A total of 136 Ti-6Al-4V square-shaped substrates (10 mm × 1 mm thickness) were used. The substrates were grit blasted with Al_2_O_3_ with particle sizes ranging from 250–500 μm (Edelkorund, Eisenbacher Dentalwaren, Wörth am Main, Germany). The grit-blasting process was performed using a sandblast machine (Renfert Basic ECO Sandblaster, Beckum, Germany) utilizing an air pressure of 5 KPa. Then, the substrates were acid-etched in a mixture of HCl (60%) and H_2_SO_4_ (70%) for 60 min at 60 °C in a hot air oven (Binder MDL 115 Drying Oven, Tuttlingen, Germany). The substrates were then rinsed in an ultrasonic bath (L&R Quantrex 90 Ultrasonic Cleaner, Kearny, NJ, USA) that contained deionized water for 20 min. The substrates were then allowed to dry in a hot air oven at 50 °C for 30 min.

Half of the SA substrates (*n* = 68) were subjected to APA using a zinc-containing BG (Zn4), and the other half were kept without abrasion. The preparation method and the nominal composition (mol.%) of the Zn4 were described elsewhere [8]. The APA of SA substrates was carried out using an air-abrasive device (LM ProPower, Parainen, Finland) with glass particle sizes ranging from 25–120 μm. Each substrate was subjected to APA for a duration of 20 s, at an angle of 90° and from a distance of 3 mm, and using air pressure of 4 KPa.

### 2.2. Simulating Body Fluid Immersion

The Zn4 air-abraded substrates (*n* = 68) were immersed in SBF for the mineralization experiments. The SBF was prepared following Kokubo’s method [33], and its chemical composition is shown in Table 1. Each Ti substrate was placed face up in a Corning^®^ 50 mL CentriStar™ Tube (Corning, Glendale, AZ, USA), and a 20 mLSBF was added. An estimation of detached Zn4 particles that detached from the Ti surface after the APA (100 mg of Zn4 per substrate) was added to each substrate and incubated in a shaking water bath (Grant LSB18; Cambridge, UK) for 14 d at 60 RPM and 37 °C. After incubation, the substrates were rinsed with ultra-pure water and allowed to dry at room temperature.

### 2.3. Surface Characterization

#### 2.3.1. Surface Roughness

A 3D non-contact optical profilometer (Bruker Nano GmbH, Billerica, MA, USA) was used to measure the surface roughness averages (Ra; arithmetical mean deviation, Rp; maximum profile peak height, Rq; root mean square deviation, Rt; total height profile, and Rv; maximum profile valley depth) for the NC-SA, Zn4-APA, and Zn4-CaP substrates. An objective lens (5×) and a multiplier (0.5) were utilized to view the substrates. The roughness average values were represented by measurements from 6 parallel substrates per group.

#### 2.3.2. Contact Angle (CA) and Surface Free Energy (SFE) Calculations

The wettability of the Zn4-CaP and NC-SA substrate surfaces was calculated by the CA method described by de Jong et al. [34]. In short, a contact angle meter (Theta, Biolin Scientific Oy, Espoo, Finland) was used to determine the CA with the sessile drop method. A droplet of distilled water or non-polar liquids is deposited on the substrate’s surface, and then 120 images are recorded in 20 s. The Young–Laplace equation was used to calculate the CA and surface tension.

The SFE was calculated on Zn4-CaP and NC-SA using the Owens–Wendt (OW) method. According to this method, the SFE (γ^tot^) of solid material comprises both a short-range polar (γ^p^) and a long-range dispersive (γ^d^) component. Three liquids, distilled ultrapure water, Diiodomethane (purity > 99%), and Formamide purity (>98%), were used as probes for SFE calculations of the substrates using the sessile drop method. The average CA and SFE values were represented by measurements from 6 parallel substrates per group.

#### 2.3.3. Scanning Electron Microscopy (SEM) and Energy Dispersive X-ray Spectroscopy (EDS) Analysis

SEM images of the NC-SA, after Zn4 abrasion (Zn4-APA), and the Zn4-CaP surfaces were carried out to evaluate the substrate’s surfaces using scanning electron microscopy (LEO Gemini 1530, Carl Zeiss, Oberkochen, Germany). In addition, EDS analysis was carried out using an X-ray detector (Thermo Scientific, Waltham, MA, USA). Three parallel substrates were used per group.

### 2.4. Antimicrobial Activity Test

The antimicrobial activity test was performed based on a method described before [8]. The antimicrobial properties of the Zn4-CaP surfaces were compared to the NC-SA surfaces. *S. mutans* Ingbritt was grown overnight in Brain Heart Infusion medium (BHI; Becton-Dickinson and Company, Sparks, MD, USA), then moved to fresh medium and allowed to grow until log-phase. The bacterial cells were then suspended in fresh BHI (A550 = 0.35), and 12 µL was pipetted onto each substrate in 24-well cell culture plates. A 10 × 10 mm Mylar film (Etra Oy, Helsinki, Finland) was used to cover each substrate and then incubated in an anaerobic chamber (Don Whitley Scientific Ltd., Shipley, UK; 80% N_2_, 10% CO_2_, 10% H_2_) at +37 °C for 4 h.

Following incubation, the film-covered substrates were immersed in 1 mL phosphate-buffered saline (PBS), and the viable bacterial cells from the substrates were harvested using micro-brushes (Quick-Stick, Dentsolv AB, Saltsjö-Boo, Sweden) and then circulated with mild sonication. The bacterial suspensions underwent serial dilution and then inoculated onto Mitis Salivarius Agar (Becton-Dickinson and Company) plates. The plates were then cultured anaerobically for 72 h at +37 °C. Bacterial colony counting was performed under a stereomicroscope, and the results were quantified as colony-forming units (CFU). Six parallel samples were used per each experimental group.

### 2.5. MC3T3-E1 Cell Culture

The Osteoblastic MC3T3-E1 assay was performed according to the method described earlier [35]. The cells were sourced from Merck Life Science Oy, Darmstadt, Germany and cultivated in α-MEM (Corning Life Sciences, Tewksbury, MA, USA) that contained 10% fetal bovine serum (FBS; Biowest, Riverside, MO, USA), 100 IU/mL penicillin and 100 µg/mL streptomycin and 24 mM Hepes buffer (Sigma-Aldrich, St. Louis, MO, USA) at +37 °C (5% CO_2_, 95% air). Prior to cell culture, the substrates were immersed in ethanol (70%) for 10 min and then allowed to dry in air. The NC-SA and Zn4-CaP substrates were then soaked in a cell culture medium for 10 min before cell seeding. A seeding density of 10,000 cells/cm^2^ was applied on the substrates or cover glasses in 24-well plates (Cellstar; Greiner Bio-One, Kremsmünster, Austria) and then cultivated for 48 h.

### 2.6. Focal Adhesion Staining

This test was conducted based on the method described earlier [35]. Twelve substrates (6 Zn4-CaP and 6 NC-SA) were used, and 6 glass slides were used as an additional control. The cells were fixed and permeabilized with 4% paraformaldehyde (PFA)-0.3% Triton X-100-PBS for 10 min. Subsequently, they were blocked with 0.2% bovine serum albumin (BSA) (Sigma-Aldrich) for 30 min at room temperature (RT). Focal adhesions of cells were stained using 1:100 diluted monoclonal anti-vinculin (Proteintech, Rosemont, IL, USA) for 1 h at RT and secondary antibody goat anti-mouse Alexa 488 (2 mg/mL stock diluted 1:100 in PBS, Invitrogen; Thermo Fisher Scientific, Waltham, MA, USA) for 1 h at RT. The actin cytoskeleton was stained with TRITC-conjugated phalloidin (0.1 mg/mL stock diluted 1:100 in PBS; Sigma-Aldrich) for 20 min at +37 °C. Nuclei were stained using Hoechst 33258 (1 mg/mL stock diluted 1:800 in PBS; Sigma-Aldrich) for 10 min at RT. The cover glasses were mounted in ImmuMount (Thermo Fisher Scientific), whereas the NC-SA and Zn4-CaP substrates were mounted in 70% glycerol-PBS. Imaging was performed using Leica Stellaris 8 Dive confocal with a DMI8 microscope (Leica, Wetzlar, Germany) and LAS X 4.5.0 acquisition software (Leica). An HC PL APO CS2 20×/0.75 DRY objective was used. The substrates were imaged with 405, 488, and 561 nm solid-state lasers with emission windows at 420–501, 501–562, and 566–706 nm, respectively. The Airy unit for the pinhole was set to 1, and the scanning speed was adjusted to 600 Hz. The image pixel size was 0.568 µm, and the images acquired with 4× zoom pixel size was 0.142 µm. Two parallel duplicates per group were used, and the test was repeated three times.

### 2.7. Cell Viability Assay with MTT

This assay is described in detail in a previous study [35]. For this test, 72 substrates (36 Zn4-CaP and 36 NC-SA) were used. Also, 36 glass slides were used as an additional control. Following a 48 h culture period, the medium was removed and replaced with a fresh medium with 0.5 mg/mL 3-[4,5-dimethylthiazol-2-yl]-2,5-diphenyl tetrazolium bromide (MTT; Sigma-Aldrich). The wells were then incubated at +37 °C for 4 h. followed by replacement of the medium with an equal volume of dimethyl sulfoxide (DMSO; Merck, Germany). Cell viability was assessed by measuring absorbances at wavelengths 550 and 650 nm (background) with Victor 2 multilabel counter (Perkin Elmer/Wallac, Turku, Finland). Cell viability on NC-SA and Zn4-CaP substrates was compared to cover glasses, which were used as controls by setting their viability to 100%. Cell viability was assessed at 1, 3, and 6 d. Four parallel replicates were used per group per time point, and the test was repeated three times.

### 2.8. Statistical Analysis

The results were represented as means and standard deviations (SD). The differences among the experimental groups were assessed using a one-way analysis of variance (ANOVA), and the statistical significance was established with *p* < 0.05. Statistical analyses were conducted using the Statistical Package for the Social Sciences [(SPSS), statistical software version 28.0 (SPSS Inc., Chicago, IL, USA)].

## 3. Results

### 3.1. Surface Roughness, Contact Angle, and Surface Free Energy

**Figure 1:**
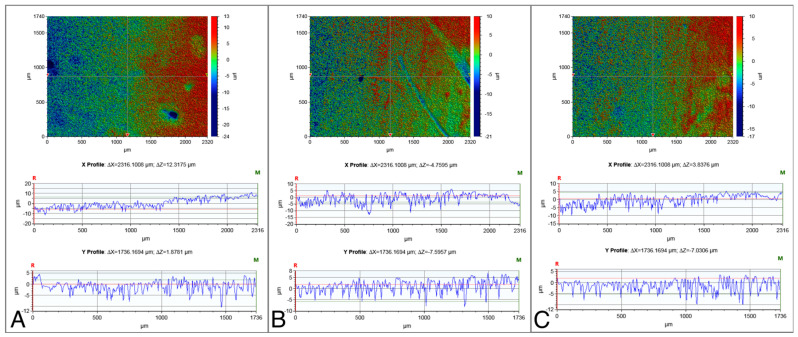
Typical surface profiles of the NC-SA (**A**), Zn4-APA (**B**), and Zn4-CaP (**C**) surfaces. The images were acquired with a 5× objective lens and utilizing a field of view multiplier of 0.5×.

The surface roughness values of the NC-SA, Zn4-APA, and Zn4-CaP are demonstrated in Table 2. Significantly lower Ra, Rp, Rq, and Rt values were characterized on the Zn4-APA and Zn4-CaP surfaces compared to the NC-SA surfaces. The Zn4-APA demonstrated a significantly lower Rv value compared to the other groups. Figure 1 shows typical surface profiles of the substrates.

The surface CA values and SFE calculations are displayed in Table 3. Zn4-CaP surfaces showed significantly lower CAs compared to the NC-SA. Furthermore, the Zn4-CaP surfaces demonstrated higher total (γ^tot^), polar (γ^p^), and dispersive (γ^d^) SFE values compared to NC-SA surfaces.

### 3.2. Scanning Electron Microscopy (SEM) and Energy Dispersive X-ray Spectroscopy (EDS) Analysis

**Figure 2:**
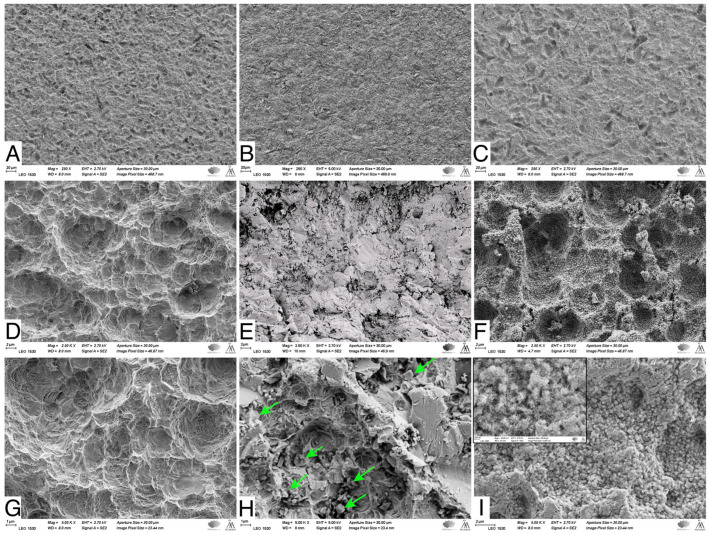
SEM images of NC-SA (**A**,**D**,**G**), Zn4-APA (**B**,**E**,**H**), and Zn4-CaP (**C**,**F**,**I**). Magnifications 250× (**A**–**C**), 2500× (**D**–**F**) and (**C**,**F**,**I**) 5000×. Arrows in (**H**) show some of the attached glass particles. The window in (**I**) shows the same picture in 25,000× magnification, illustrating the possible HA crystals.

SEM images showed that the NC-SA demonstrated macro- and micro-surface roughness resulting from the sandblasting and acid-etching processes (Figure 2A,D,G). The Zn4 APA led to an even surface topography due to the BG particles trapped in the SA irregularities (Figure 2B,E,H). In contrast, after the SBF immersion, the Zn4-CaP surfaces were mineralized with a CaP layer covering the entire surface (Figure 2C,F,I), which was verified by the EDS analysis that showed the presence of Ca/P at 2.2 molar ratio (Table 4). Additionally, the large amount of Si and the absence of Na suggest that the coating consisted of a silica-rich layer and CaP crystals. Also, Zn was present in the coating. However, the SEM analyses cannot verify whether Zn was incorporated in the CaP layer structure or the silica-rich layer. Conversely, on NC-SA surfaces, mainly Ti was detected (Table 4).

### 3.3. Antibacterial Effect

**Figure 3:**
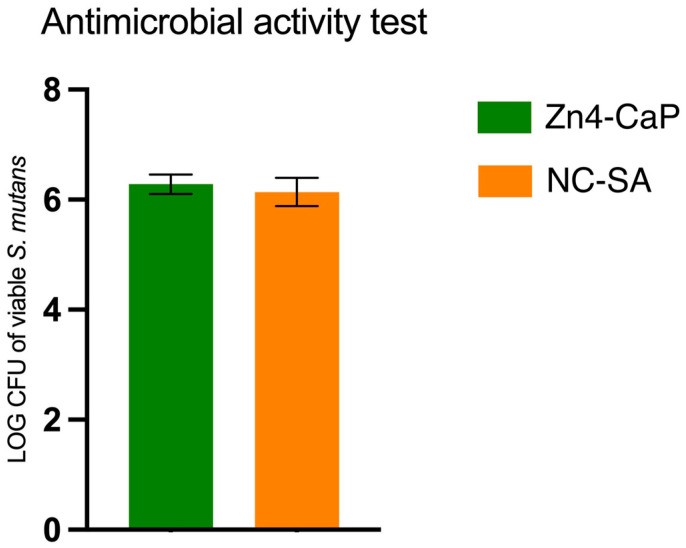
Viable *S. mutans* on the Zn4-CaP and NC-SA substrates.

The viability of *S. mutans* was studied on Zn4-CaP and NC-SA, and the result was expressed in a log–colony-forming unit (CFU). The results demonstrated that the *S. mutans* viability was not significantly different between the substrates (Figure 3).

### 3.4. Focal Adhesion and MTT Results

**Figure 4:**
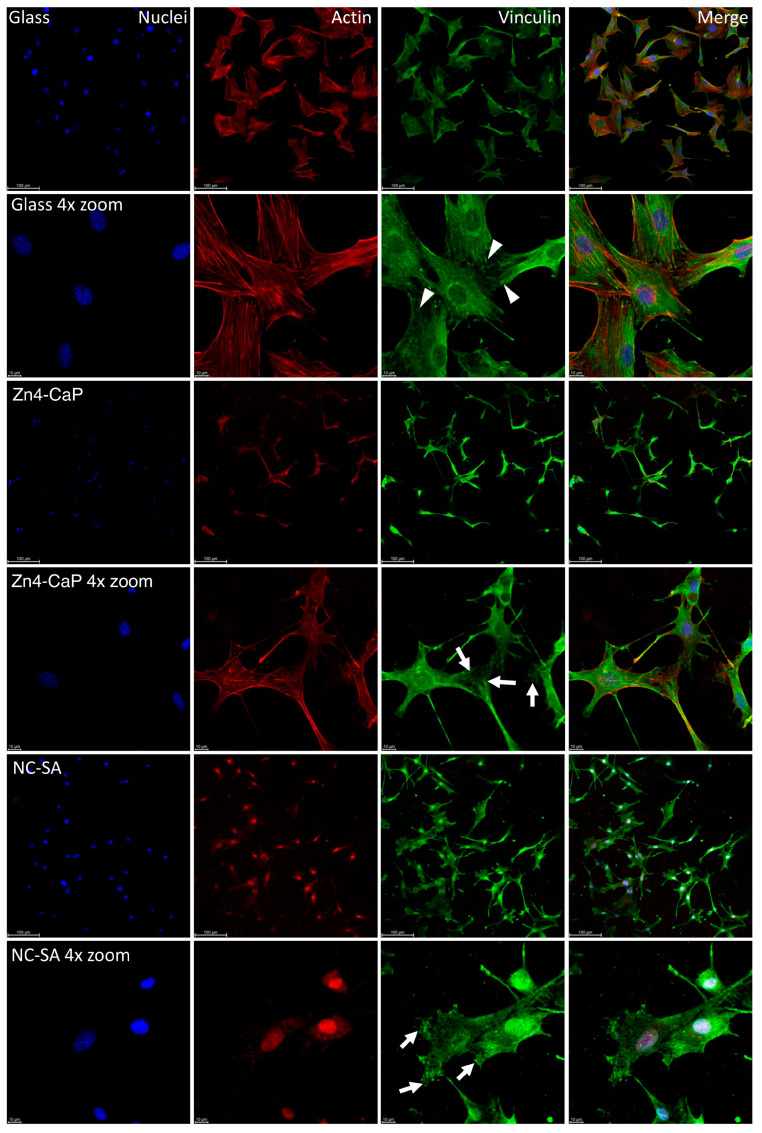
Localization of focal adhesion in MC3T3-E1 cells. Focal adhesion staining of the cells on cover glass, Zn4-CaP, and NC-SA after 48 h culture (images presented here represent one culture). Hoechst 33258 was used for nucleus staining (blue), anti-vinculin was employed for focal adhesion labeling (green), and TRITC-phalloidin was utilized for visualizing the actin cytoskeleton (red). A fluorescence microscope was used to capture the images with 20× objective (with or without 4× zoom). The focal adhesion-like structures were observed at the peripheries of cells on all surfaces. On cover glasses, these structures were thin dash-like structures (arrowheads), whereas, on Zn4-CaP and NC-SA, the structures were more spread over the surface of the substrate (arrows). On cover glass and Zn4-CaP, the actin cytoskeleton of the cells is normal, but on NC-SA, actin localizes mainly in nuclei, and only a small amount is visible in the cytoplasm.

The vinculin staining showed focal adhesion on all substrates, represented by small dot-like structures on the cell peripheries. On the cover glass, the focal adhesions were thin dash-like structures, whereas, on Zn4-CaP and NC-SA, the structures were more spread over the substrate surfaces. Furthermore, the actin cytoskeleton of the cells is normal on the cover glass and Zn4-CaP substrates, but on NC-SA, only a small amount of actin is visible in the cytoplasm and is mainly localized in nuclei (Figure 4).

**Figure 5:**
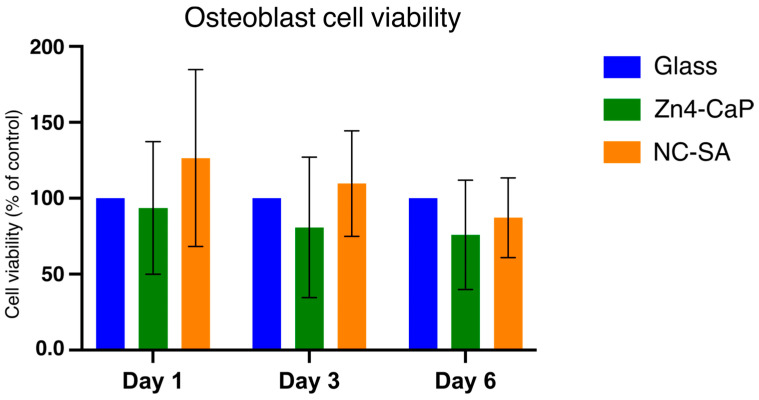
MTT assay results of MC3T3-E1 cells cultured on cover glass, Zn4-CaP, and NC-SA. Cell viability on Zn4-CaP and NC-SA substrates was assessed relative to cover glasses, serving as controls with their viability set to 100%. The data represent the mean (SD) pooled from three independent cell cultures.

The viability of MC3T3-E1 was tested on the Zn4-CaP. The MTT assay indicated that the cell viability on the Zn4-CaP surfaces at all time points was not significantly different from that on the NC-SA or the cover glasses (Figure 5).

## 4. Discussion

The results of this study demonstrated that APA using Zn4 induces CaP layer formation on the SA Ti surface in simulated body conditions. Furthermore, this study also shows that the viability of MC3T3 cells on Zn4-CaP is comparable to that on NC-SA or cover glass. Nevertheless, the mineralized Zn4-CaP layer did not show antibacterial effects against S. mutans compared to the NC-SA. Our earlier work demonstrated that BG APA of Ti can alter the abraded surfaces, leading to firm attachment of glass particles to these surfaces. This finding was further validated through a 15 min ultrasonic bath treatment of the abraded Ti substrates, which did not impact the amount of the adhered glass particles, as verified by SEM-EDS analysis [8]. In a clinical context, following the decontamination of implant surface biofilm by APA, the detached abrasive materials remain in the vicinity of the peri-implant sulcus. To replicate this in the current investigation, an estimation of the glass particles detached during the APA process (100 mg of Zn4 per substrate) was incorporated into the SBF.

Zn4 APA has been shown to impart strong antibacterial effects to abraded Ti surfaces [8]. In addition, APA has demonstrated effectiveness in decontaminating bacterial biofilms formed on SA Ti surfaces in vitro [9,10], and in vivo [36]. These findings indicate that BG APA has promising potential in treating PI by decontaminating implant surface biofilm. Further, the attached BG particles yield an extended antibacterial effect on the surface, potentially producing an HA layer. However, the current study demonstrated that the mineralization of the Ti surface by the Zn4-CaP layer resulted in a diminished antibacterial effect against *S. mutans*. The limited antibacterial effect can be explained by the low amount of unreacted glass left from the BG particles after the formation and mineralization of the Ca-P layer [37,38]. Although Zn4 has a lower dissolution rate than 45S5 BG, the SBF incubation time was long enough to convert the glass particles almost entirely into CaP. Consequently, this led to negligible ion release, thus limiting the antibacterial effect. It should be emphasized that the measurement of ion dissolution from the Zn4-CaP layer was not conducted. This aspect, along with the exploration of shorter SBF immersion times, will be considered in future research.

Previous research has indicated that the Zn^2+^ ion has strong antibacterial properties against various clinically important pathogens [39,40,41]. Furthermore, it has a vital role in osteoblast cell proliferation and differentiation, thus promoting bone healing [42,43]. The results suggest that Zn in the surface layer was incorporated into the HA structure or dissolved in too-low concentrations to provide an enhanced antibacterial effect for the Zn4-abraded and SBF-treated samples.

The surface roughness influences the proliferation and differentiation of osteoblastic cells [44]. Macro- and micro-surface roughness produced by the SA process has led to higher osteoblast activity than on machined surfaces [45]. However, Cho et al. [46] reported somehow contradicting results that the osteoblast cell proliferation rate was slightly higher on machined compared to SA surface. They also showed a superior osteogenic response on the SA surface as expressed by elevated alkaline phosphatase activities and higher gene expression bone marker levels. In our study, the Zn4-CaP coating resulted in lower surface roughness values than the SA surfaces. Despite the decrease in surface roughness, the osteoblast attachment and viability were not significantly different from that on SA surfaces. The nanosized crystals of the Zn4-CaP likely provide suitable nano- and microroughness for osteoblast proliferation.

It is well-established that the surface roughness of Ti influences wettability and SFE components (polar and dispersive) [47]. Cell attachment and differentiation are significantly affected by these physicochemical properties [48,49,50,51]. The surface wettability, determined by SFE, is also a crucial factor that affects cellular behavior [39]. SFE value with a high polar component reflects low CA and higher surface wettability and, therefore, enhances interaction with the surrounding biological environment [52]. Increasing wettability can promote the spreading and adhesion of matrix proteins, thereby promoting the attachment of cells [53]. Latifi et al. found that altering the surface wettability of Ti is more crucial than roughness. They demonstrated that surfaces with higher wettability exhibit greater cell attachment compared to surfaces than those with lower wettability [54]. However, conflicting results have also been reported and showed that the increased surface wettability has no effect or negative effect on cellular behavior [55,56]. Yet, in this study, despite the good wettability on the Zn4-CaP surfaces, the osteoblast cell viability was not significantly different from that on the NC-SA.

Immunofluorescence staining was used in the current study to identify the focal adhesion of cells on the substrate’s surfaces. Vinculin immunofluorescence of the cells cultured on Zn4-CaP and NC-SA substrates showed thin focal adhesion with similar cell morphology, indicating that the coating is biocompatible and does not adversely affect cell viability. These results agree with a previous study that evaluated the same cell line on the HA layer made by atomic layer deposition [35]. Conversely, when MC3T3-E1 cells were cultured on freshly BG air-abraded surfaces, significantly higher cell viability was observed in the first 24 h of culturing on BG-abraded substrates compared to the SA surface, as demonstrated in a previous study [11]. Yet, the same study showed no difference in viability when culture duration is prolonged to 3 or 6 d [11]. The authors hypothesize that higher ion release, such as Si, Ca, and Zn, from the BG in the early time of cell culture, could promote osteoblast cell viability [11,42,43,57,58].

The mineralized Ca-P surface layer developed during SBF immersion retards the ion dissolution from the unreacted glass particles [34,35]. Kobayashi and his co-workers [59] investigated the behavior of MC3T3-E1 on HA-dispersed Ti-based composite plates with different HA/Ti ratios. Their findings have been inconsistent with our results, and they reported a decrease in the adhesion and proliferation of osteoblast cells with an increased HA ratio. It is noteworthy that, in this study, the Ca/P molar ratio achieved on Ti substrates following immersion in SBF is 2.2. Given that the Ca/P molar ratio of HA is 1.67 [60,61], the ratio 2.2 given by the SEM-EDS analysis suggests enrichment of Ca and P species on the surface, and it is anticipated that the crystals of the Zn4-CaP layer would exhibit characteristics like HA crystals. Therefore, in-depth research with diverse abrasive glass particle sizes and varied SBF immersion times to produce different Ca/P ratios is needed to investigate how the coating composition affects its osteogenic properties. In addition, a detailed study of ion dissolution from the Zn4-CaP is required to assess the true potential of the antimicrobial effect of the coating. Notably, the ratio of unreacted glass to CaP must be optimal to provide high enough concentrations of dissolved ionic species in the interfacial solution for a desired antibacterial effect. In general, in vitro studies have limitations because they do not represent the complexity of the in vivo environment. In this context, the SBF immersion conditions do not necessarily represent the peri-implant tissue interface.

## 5. Conclusions

This study indicates that BG APA can modify SA Ti surfaces to form a calcium phosphate coating. The coating can decrease the surface roughness, enhance wettability, and support the attachment and viability of osteoblast cells. However, the coating did not impart an extra antibacterial effect against *S. mutans* over the non-coated surface. Considering the limitations of the current study, it can be concluded that APA has promising potential for the treatment of peri-implantitis. In future research, ion dissolution from the Zn4-CaP layer will be evaluated at different immersion durations. Additionally, Fourier transform infrared spectroscopy-attenuated total reflectance and X-ray diffraction analysis will be used to provide more insights into the characteristics of the CaP layer.

## Figures and Tables

**Table 1:** Composition of simulated body fluid (g/L) according to Kokubo et al. [33].

Component	Concentration (g/L)
NaCl	7.996
NaHCO_3_	0.35
KCl	0.224
K_2_HPO_4_·3H_2_O	0.228
MgCl_2_·6H_2_O	0.305
CaCl_2_	0.278
Na_2_SO_4_	0.071
(CH_2_OH)_3_CNH_2_	6.057

**Table 2:** Mean (SD) of surface roughness values of the non-coated sandblasted and acid-etched surface (NC-SA) after air abrasion with zinc-containing BG (Zn4-APA) and after the formation of zinc-containing calcium phosphate coating (Zn4-CaP).

Substrate	Ra	Rp	Rq	Rt	Rv
NC-SA	3.695 (0.086)	12.71 (0.64)	4.53 (0.31)	37.70 (3.90)	−24.98 (4.22)
Zn4-APA	2.526 (0.054) ***	9.23 (0.39) ***	3.37 (0.14) ***	27.64 (0.90) ***	−18.33 (0.95) *
Zn4-CaP	2.565 (0.042) ***	10.79 (1.58) ***	3.52 (0.31) ***	31.12 (3.73) **	−20.71 (3.89)

Ra; arithmetical mean deviation, Rp; maximum profile peak height, Rq; root mean square deviation, Rt; total height of profile, and Rv; maximum profile valley depth. * *p* = 0.07, ** *p* < 0.01, and *** *p* < 0.001.

**Table 3:** Mean (SD) of water and non-polar liquids contact angle values and surface free energy calculations on the NC-SA and Zn4-CaP substrate’s surfaces. *** *p* < 0.001.

Substrate	Contact Angles CA (°)			Surface Free Energy (SFE)		
	Water	Diiodomethane	Formamide	Total (γ^tot^)	Dispersive (γ^D^)	Polar (γ^P^)
NC-SA	77.19 (4.31)	64.48 (3.32)	40.73 (3.01)	40.43 (1.14)	30.14 (2.08)	10.38 (1.46)
Zn4-CaP	13.35 (1.58) ***	11.06 (0.86) ***	9.57 (1.20) ***	71.13 (0.22) ***	43.88 (0.14) ***	27.22 (0.21) ***

**Table 4:** EDS analysis of the surface chemical composition of NA-SA, Zn4-APA, and Zn4-CaP.

Substrate		Na	Al	Ti	Cr	Cl	Si	P	Ca	Zn
NC-SA	Weight %	0.44	5.69	92.51	0.88	0.17				
	Atom %	0.87	9.63	88.25	0.77	0.22				
Zn4-APA	Weight %	5.76	2.49	45.35	0.38	0.32	3.27	0.13	2.39	0.54
	Atom %	6.32	2.33	23.87	0.18	0.27	2.94	0.11	1.51	0.21
Zn4-CaP	Weight %		0.63	18.73	0.19	0.49	15.46	3.32	9.48	3.22
	Atom %		0.53	8.88	0.11	0.31	12.50	2.43	5.37	1.12

## Data Availability

Data are available from the corresponding authors upon reasonable request.

## References

[1] Schwarz F., Derks J., Monje A., Wang H.L. (2018). Peri-implantitis. J. Periodontol..

[2] Suarez F., Monje A., Galindo-Moreno P., Wang H.L. (2013). Implant surface detoxification: A comprehensive review. Implant. Dent..

[3] Lee S.W., Phillips K.S., Gu H., Kazemzadeh-Narbat M., Ren D. (2021). How microbes read the map: Effects of implant topography on bacterial adhesion and biofilm formation. Biomaterials.

[4] Estefanía-Fresco R., García-de-la-Fuente A.M., Egaña-Fernández-Valderrama A., Bravo M., Aguirre-Zorzano L.A. (2019). One-year results of a nonsurgical treatment protocol for peri-implantitis. A retrospective case series. Clin. Oral Implants Res..

[5] Heitz-Mayfield L.J.A., Salvi G.E. (2018). Peri-implant mucositis. J. Clin. Periodontol..

[6] Keim D., Nickles K., Dannewitz B., Ratka C., Eickholz P., Petsos H. (2019). In vitro efficacy of three different implant surface decontamination methods in three different defect configurations. Clin. Oral Implants Res..

[7] Moharrami M., Perrotti V., Iaculli F., Love R., Quaranta A. (2019). Effects of air abrasive decontamination on titanium surfaces: A systematic review of in vitro studies. Clin. Implant Dent. Relat. Res..

[8] Abushahba F., Söderling E., Aalto-Setälä L., Sangder J., Hupa L., Närhi T.O. (2018). Antibacterial properties of bioactive glass particle abraded titanium against Streptococcus mutans. Biomed. Phys. Eng. Express.

[9] Abushahba F., Söderling E., Aalto-Setälä L., Hupa L., Närhi T.O. (2019). Air Abrasion with Bioactive Glass Eradicates Streptococcus mutans Biofilm From a Sandblasted and Acid-Etched Titanium Surface. J. Oral Implantol..

[10] Abushahba F., Gürsoy M., Hupa L., Närhi T.O. (2021). Effect of bioactive glass air-abrasion on Fusobacterium nucleatum and Porphyromonas gingivalis biofilm formed on moderately rough titanium surface. Eur. J. Oral Sci..

[11] Abushahba F., Tuukkanen J., Aalto-Setälä L., Miinalainen I., Hupa L., Närhi T.O. (2020). Effect of bioactive glass air-abrasion on the wettability and osteoblast proliferation on sandblasted and acid-etched titanium surfaces. Eur. J. Oral Sci..

[12] Allan I., Newman H., Wilson M. (2001). Antibacterial activity of particulate bioglass against supra- and subgingival bacteria. Biomaterials.

[13] Stoor P., Söderling E., Salonen J.I. (1998). Antibacterial effects of a bioactive glass paste on oral microorganisms. Acta Odontol. Scand..

[14] Begum S., Johnson W.E., Worthington T., Martin R.A. (2016). The influence of pH and fluid dynamics on the antibacterial efficacy of 45S5. Bioglass. Biomed. Mater..

[15] Allan I., Newman H., Wilson M. (2002). Particulate Bioglass reduces the viability of bacterial biofilms formed on its surface in an in vitro model. Clin. Oral Implants Res..

[16] Hammami I., Gavinho S.R., Jakka S.K., Valente M.A., Graça M.P.F., Pádua A.S., Silva J.C., Sá-Nogueira I., Borges J.P. (2023). Antibacterial Biomaterial Based on Bioglass Modified with Copper for Implants Coating. J. Funct. Biomater..

[17] Drago L., Toscano M., Bottagisio M. (2018). Recent Evidence on Bioactive Glass Antimicrobial and Antibiofilm Activity: A Mini-Review. Materials.

[18] Hench L.L. (1991). Bioceramics: From Concept to Clinic. J. Am. Ceram. Soc..

[19] Sinitsyna P., Karlström O., Sevonius C., Hupa L. (2022). In vitro dissolution and characterisation of flame-sprayed bioactive glass microspheres S53P4 and 13–93. J. Non-Cryst. Solids.

[20] Deng Y., Chen W., Li B., Wang C., Kuang T., Li Y. (2020). Physical vapor deposition technology for coated cutting tools: A review. Ceram. Int..

[21] Huang C.H., Yoshimura M. (2020). Direct ceramic coating of calcium phosphate doped with strontium via reactive growing integration layer method on α-Ti alloy. Sci. Rep..

[22] Gross K.A., Berndt C.C., Herman H. (1998). Amorphous phase formation in plasma-sprayed hydroxyapatite coatings. J. Biomed. Mater. Res..

[23] Heimann R.B. (2016). Plasma-Sprayed Hydroxylapatite-Based Coatings: Chemical, Mechanical, Microstructural, and Biomedical Properties. J. Therm. Spray Technol..

[24] Nahum E.Z., Lugovskoy S., Lugovskoy A., Kazanski B., Sobolev A. (2023). The study of hydroxyapatite growth kinetics on CP—Tiand Ti65Zr treated by Plasma electrolytic oxidation process. J. Mater. Res. Technol..

[25] Astaneh S.H., Faverani L.P., Sukotjo C., Takoudis C.G. (2021). Atomic layer deposition on dental materials: Processing conditions and surface functionalization to improve physical, chemical, and Clinical Properties—A Review. Acta Biomater..

[26] Akhtach S., Tabia Z., Bricha M., El Mabrouk K. (2021). Structural characterization, in vitro bioactivity, and antibacterial evaluation of low silver-doped bioactive glasses. Ceram. Int..

[27] Luo S.H., Xiao W., Wei X.J., Jia W.T., Zhang C.Q., Huang W.H., Jin D.X., Rahaman M.N., Day D.E. (2010). In vitro evaluation of cytotoxicity of silver-containing borate bioactive glass. J. Biomed. Mater. Res. Part B Appl. Biomater..

[28] Mohd Bakhori S.K., Mahmud S., Ling C.A., Sirelkhatim A.H., Hasan H., Mohamad D., Masudi S.M., Seeni A., Abd Rahman R. (2017). In-vitro efficacy of different morphology zinc oxide nanopowders on Streptococcus sobrinus and Streptococcus mutans. Mater. Sci. Eng. C Mater. Biol. Appl..

[29] Ciosek Ż., Kot K., Rotter I. (2023). Iron, Zinc, Copper, Cadmium, Mercury, and Bone Tissue. Int. J. Environ. Res. Public Health.

[30] Yu Y., Liu K., Wen Z., Liu W., Zhang L., Su J. (2020). Double-edged effects and mechanisms of Zn^2+^microenvironments on osteogenic activity of BMSCs: Osteogenic differentiation or apoptosis. RSC Adv..

[31] Costa M.I., Sarmento-Ribeiro A.B., Gonçalves A.C. (2023). Zinc: From Biological Functions to Therapeutic Potential. Int. J. Mol. Sci..

[32] Koller G., Cook R.J., Thompson I.D., Watson T.F., Di Silvio L. (2007). Surface modification of titanium implants using bioactive glasses with air abrasion technologies. J. Mater. Sci. Mater. Med..

[33] Kokubo T., Kushitani H., Sakka S., Kitsugi T., Yamamuro T. (1990). Solutions able to reproduce in vivo surface-structure changes in bioactive glass-ceramic A-W. J. Biomed. Mater. Res..

[34] de Jong H.P., van Pelt A.W., Arends J. (1982). Contact angle measurements on human enamel—An in vitro study of influence of pellicle and storage period. J. Dent. Res..

[35] Kylmäoja E., Holopainen J., Abushahba F., Ritala M., Tuukkanen J. (2022). Osteoblast Attachment on Titanium Coated with Hydroxyapatite by Atomic Layer Deposition. Biomolecules.

[36] Abushahba F., Areid N., Gürsoy M., Willberg J., Laine V., Yatkin E., Hupa L., Närhi T.O. (2023). Bioactive glass air-abrasion promotes healing around contaminated implant surfaces surrounded by circumferential bone defects: An experimental study in the rat. Clin. Implant Dent. Relat. Res..

[37] Aalto-Setälä L., Siekkinen M., Lindfors N., Hupa L. (2023). Dissolution of Glass–Ceramic Scaffolds of Bioactive Glasses 45S5 and S53P4. Biomed. Mater. Devices.

[38] Siekkinen M., Karlström O., Hupa L. (2022). Effect of local ion concentrations on the in vitro reactions of bioactive glass 45S5 particles. Int. J. Appl. Glass Sci..

[39] Krishnamoorthy R., Athinarayanan J., Periyasamy V.S., Alshuniaber M.A., Alshammari G., Hakeem M.J., Ahmed M.A., Alshatwi A.A. (2022). Antibacterial Mechanisms of Zinc Oxide Nanoparticle against Bacterial Food Pathogens Resistant to Beta-Lactam Antibiotics. Molecules.

[40] Hamouda R.A., Alharbi A.A., Al-Tuwaijri M.M., Makharita R.R. (2023). The Antibacterial Activities and Characterizations of Biosynthesized Zinc Oxide Nanoparticles, and Their Coated with Alginate Derived from Fucus vesiculosus. Polymers.

[41] Wei Y., Wang J., Wu S., Zhou R., Zhang K., Zhang Z., Liu J., Qin S., Shi J. (2022). Nanomaterial-Based Zinc Ion Interference Therapy to Combat Bacterial Infections. Front. Immunol..

[42] Yusa K., Yamamoto O., Takano H., Fukuda M., Iino M. (2016). Zinc-modified titanium surface enhances osteoblast differentiation of dental pulp stem cells in vitro. Sci. Rep..

[43] Seo H.J., Cho Y.E., Kim T., Shin H.I., Kwun I.S. (2010). Zinc may increase bone formation through stimulating cell proliferation, alkaline phosphatase activity and collagen synthesis in osteoblastic MC3T3-E1 cells. Nutr. Res. Pract..

[44] Martinez M.A.F., Balderrama Í.F., Karam P.S.B.H., de Oliveira R.C., de Oliveira F.A., Grandini C.R., Vicente F.B., Stavropoulos A., Zangrando M.S.R., Sant’Ana A.C.P. (2020). Surface roughness of titanium disks influences the adhesion, proliferation and differentiation of osteogenic properties derived from human. Int. J. Implant Dent..

[45] Velasco-Ortega E., Fos-Parra I., Cabanillas-Balsera D., Gil J., Ortiz-García I., Giner M., Bocio-Núñez J., Montoya-García M.J., Jiménez-Guerra Á. (2023). Osteoblastic Cell Behavior and Gene Expression Related to Bone Metabolism on Different Titanium Surfaces. Int. J. Mol. Sci..

[46] Cho Y.D., Kim W.J., Kim S., Ku Y., Ryoo H.M. (2021). Surface Topography of Titanium Affects Their Osteogenic Potential through DNA Methylation. Int. J. Mol. Sci..

[47] Elias C.N., Oshida Y., Lima J.H., Muller C.A. (2008). Relationship between surface properties (roughness, wettability and morphology) of titanium and dental implant removal torque. J. Mech. Behav. Biomed. Mater..

[48] Pegueroles M., Aparicio C., Bosio M., Engel E., Gil F.J., Planell J.A., Altankov G. (2010). Spatial organization of osteoblast fibronectin matrix on titanium surfaces: Effects of roughness, chemical heterogeneity and surface energy. Acta Biomater..

[49] Abrahamsson I., Zitzmann N.U., Berglundh T., Linder E., Wennerberg A., Lindhe J. (2002). The mucosal attachment to titanium implants with different surface characteristics: An experimental study in dogs. J. Clin. Periodontol..

[50] Kohavi D., Badihi Hauslich L., Rosen G., Steinberg D., Sela M.N. (2013). Wettability versus electrostatic forces in fibronectin and albumin adsorption to titanium surfaces. Clin. Oral Implants Res..

[51] Ostrovskaya L., Perevertailo V., Ralchenko V., Dementjev A., Loginova O. (2002). Wettability and surface energy of oxidized and hydrogen plasma-treated diamond films. Diam. Relat. Mater..

[52] Bociaga D., Sobczyk-Guzenda A., Komorowski P., Balcerzak J., Jastrzebski K., Przybyszewska K., Kaczmarek A. (2019). Surface Characteristics and Biological Evaluation of Si-DLC Coatings Fabricated Using Magnetron Sputtering Method on Ti6Al7Nb Substrate. Nanomaterials.

[53] Dohan Ehrenfest D.M., Coelho P.G., Kang B.S., Sul Y.T., Albrektsson T. (2010). Classification of osseointegrated implant surfaces: Materials, chemistry and topography. Trends Biotechnol..

[54] Latifi S., Shankar R., Donahue H. (2020). Polydopamine Coating on Titanium Affects Osteoblastic Differentiation to a Greater Degree than Does Surface Roughness. Adv. Mater. Phys. Chem..

[55] Gittens R.A., Scheideler L., Rupp F., Hyzy S.L., Geis-Gerstorfer J., Schwartz Z., Boyan B.D. (2014). A review on the wettability of dental implant surfaces II: Biological and clinical aspects. Acta Biomater..

[56] Eriksson C., Nygren H., Ohlson K. (2004). Implantation of hydrophilic and hydrophobic titanium discs in rat tibia: Cellular reactions on the surfaces during the first 3 weeks in bone. Biomaterials.

[57] Foppiano S., Marshall S.J., Marshall G.W., Saiz E., Tomsia A.P. (2007). Bioactive glass coatings affect the behavior of osteoblast-like cells. Acta Biomater..

[58] Xynos I.D., Hukkanen M.V., Batten J.J., Buttery L.D., Hench L.L., Polak J.M. (2000). Bioglass 45S5 stimulates osteoblast turnover and enhances bone formation In vitro: Implications and applications for bone tissue engineering. Calcif. Tissue Int..

[59] Kobayashi M., Nihonmatsu S., Okawara T., Onuki H., Sakagami H., Nakajima H., Takeishi H., Shimada J. (2019). Adhesion and Proliferation of Osteoblastic Cells on Hydroxyapatite-dispersed Ti-based Composite Plate. In Vivo.

[60] Guo H., Wei J., Yuan Y., Liu C. (2007). Development of calcium silicate/calcium phosphate cement for bone regeneration. Biomed. Mater..

[61] Raynaud S., Champion E., Bernache-Assollant D., Thomas P. (2002). Calcium phosphate apatites with variable Ca/P atomic ratio I. Synthesis, characterisation and thermal stability of powders. Biomaterials.

